# Velvet Antler Peptides Reduce Scarring *via* Inhibiting the TGF-β Signaling Pathway During Wound Healing

**DOI:** 10.3389/fmed.2021.799789

**Published:** 2022-01-21

**Authors:** Guokun Zhang, Dongxu Wang, Jing Ren, Hongmei Sun, Jiping Li, Shengnan Wang, Liyan Shi, Zhen Wang, Mengjie Yao, Haiping Zhao, Chunyi Li

**Affiliations:** ^1^Institute of Special Animal and Plant Sciences, Chinese Academy of Agricultural Sciences (CAAS), Changchun, China; ^2^Institute of Antler Science and Product Technology, Changchun Sci-Tech University, Changchun, China; ^3^Jilin Academy of Sika Deer Industry, Changchun, China; ^4^Key Laboratory of Antler Biology of Jilin, Changchun, China; ^5^Department of Pathology, China-Japan Union Hospital, Jilin University, Changchun, China; ^6^College of Animal Science and Technology, Qingdao Agricultural University, Qingdao, China

**Keywords:** velvet antler, peptides, wound healing, scar, transforming growth factor-β

## Abstract

**Aim:**

Scar formation generally occurs in cutaneous wound healing in mammals, mainly caused by myofibroblast aggregations, and currently with few effective treatment options. However, the pedicle wound (about 10 cm in diameter) of the deer can initiate regenerative healing, which has been found to be achieved *via* paracrine factors from the internal tissues of antlers.

**Methods:**

Enzymatically digested velvet antler peptides (EVAP) were prepared along with other types of antler extracts as the controls. The effects of EVAP on healing of full-thickness skin wounds were evaluated using rats *in vivo*, and on myofibroblast transdifferentiation tested using transforming growth factor-β1 (TGF-β1)-induced human dermal fibroblasts *in vitro*.

**Results:**

EVAP significantly accelerated the wound healing rate, reduced scar formation, and improved the healing quality, including promoted angiogenesis, increased number of skin appendages (hair follicles and sebaceous glands) and improved the distribution pattern of collagen fibers (basket-wave like) in the healed tissue. Moreover, EVAP significantly down-regulated the expression levels of genes pro- scar formation (Col1a2 and TGF-β1), and up-regulated the expression levels of genes anti-scar formation (Col3a1 and TGF-β3), and suppressed the excessive transdifferentiation of myofibroblasts and the formation of collagen I *in vivo* and *in vitro*. Furthermore, we found these effects were highly likely achieved by inhibiting the TGF-β signaling pathway, evidenced by decreased expression levels of the related genes, including TGF-β1, Smad2, p-Smad2, α-SMA, and collagen I.

**Conclusions:**

EVAP may be a promising candidate to be developed as a clinic drug for regenerative wound healing.

## Introduction

Wound healing in mammals generally leads to scar formation, which causes psychological and physiological distress in humans. Therefore, great efforts have been invested in improving wound healing quality in clinics, particularly reducing scar formation (1). Scars are composed of excessive collagen I fibers in thick bundles and intermingled with myofibroblasts that exert contraction during the process of wound healing (2, 3). Myofibroblasts are known as collagen secreting cells that have the contractile ability and form microfilament bundles (2, 3). According to the report that myofibroblasts are mainly transdifferentiated from fibroblasts (4, 5); and this transdifferentiation relies on the transforming growth factor-β (TGF-β) signaling pathway (6–8). After wounding, both activated fibroblasts and infiltrated immune cells, such as macrophages, start to express TGF-β1, which binds to TGF-β receptors of fibroblasts (9). This binding complex initiates the TGF-β signaling pathway and induces myofibroblast transdifferentiation. Currently, intervention of the TGF-β signaling pathway may be an effective way to inhibit myofibroblast transdifferentiation, are proposed therapeutic approaches to reduce scar formation and even achieve scarless wound healing.

Deer antlers are the only mammalian organs capable of complete renewal from the permanent bony protuberances, known as pedicles (10, 11). Full antler regeneration initiates from the essentially scarless wound healing over the apex of a pedicle (12–15). Studies show that this scarless wound healing depends entirely on the closely associated with internal antler tissue, in which antler stem cell (AnSC) reside (14, 15). Recently, our group found that α-smooth muscle actin (α-SMA) was almost absent in the healing skin over pedicle wounds; at the same time, the TGF-β signaling pathway was significantly down-regulated in the healing skin (16). Generally, the presence of α-SMA has been considered a marker for myofibroblast differentiation, which is usually induced by the TGF-β signaling pathway (17, 18). We speculate that this regenerative wound healing may be achieved by the paracrine factors of the AnSC-derived antler tissue, and these factors can be extracted from the antler tissue. Indeed, some previous studies have demonstrated that antler extracts could effectively treat fibrotic diseases through intragastric administration, and these diseases include carbon tetrachloride-induced liver fibrosis (19), pressure overload-induced cardiac fibrosis (20), and bleomycin-induced pulmonary fibrosis (21), suggesting that the putative paracrine factors can be not only be extractable, but also digestive enzymes (mainly pepsin and trypsin) may be involved in the production of the potent bioactive polypeptides from the administered antler extracts.

Here, we evaluated the therapeutic effects of enzymatically digested velvet antler peptides (EVAP) on scarring in wound healing. We found that EVAP could improve healing quality (reduction of scar formation) of full-thickness skin wounds in rats and inhibit the transdifferentiation from fibroblasts to myofibroblasts, which may achieve through the intervention of the TGF-β signaling pathway. We believe our study will provide a novel strategy for reducing scar formation and even realizing scarless wound healing in a clinical setting.

## Materials and Methods

### Preparation of EVAP

**Figure 1:**
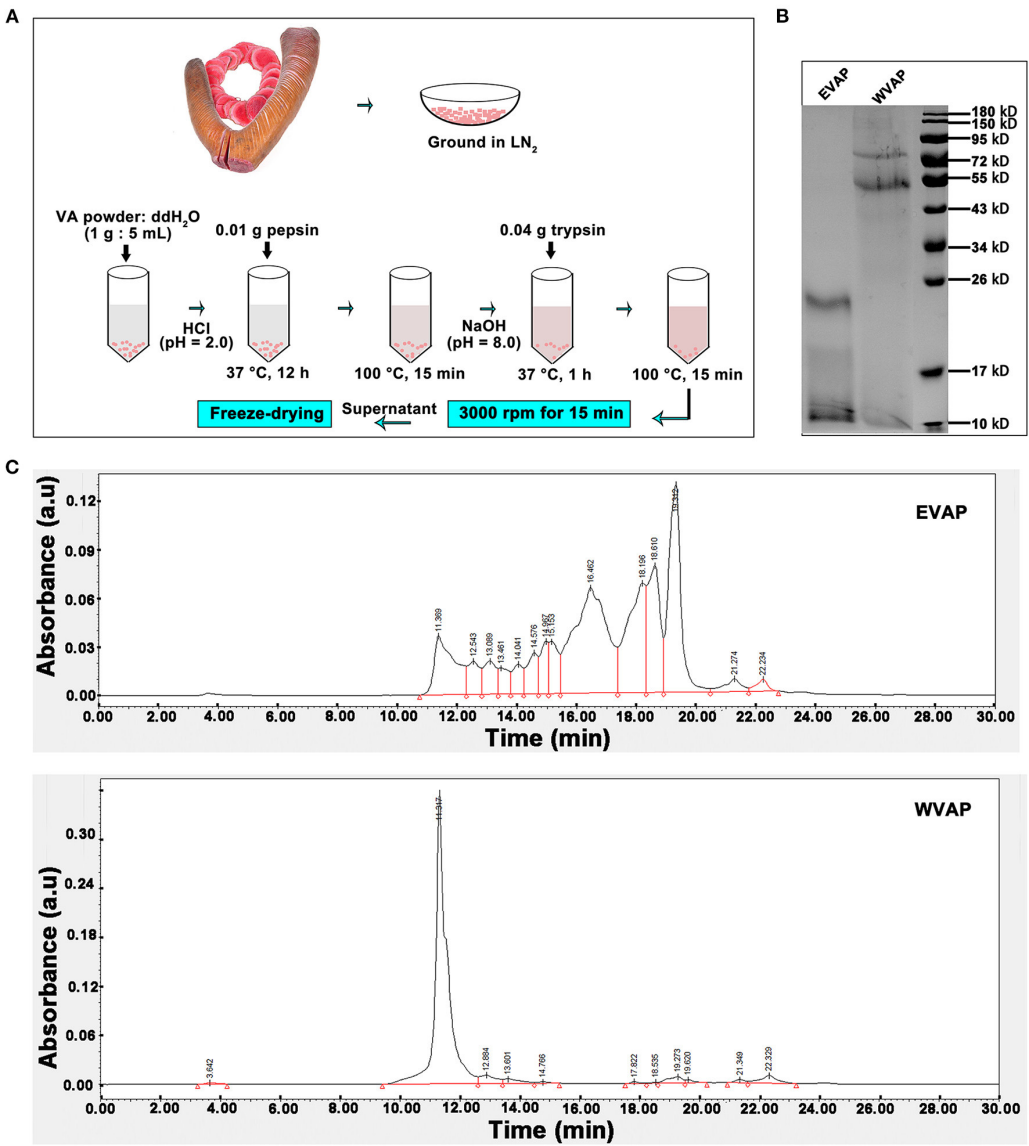
Preparation and characterization of EVAP. **(A)** Schematic drawing of EVAP preparation procedure. **(B)** SDS-PAGE electrophoresis of EVAP and WVAP. Note that molecular weight of the EVAP lies between 10 and 26 kD. **(C)** Profiling of EVAP and WVAP through HPLC. Note that compared to WVAP, the profiling of EVAP had shifted toward the lower molecular end and consisted of 14 peaks. EVAP, enzymatically-digested velvet antler peptides; HPLC, high performance liquid chromatography; LN2, liquid nitrogen; SDS-PAGE, sodium dodecyl sulfate-polyacrylamide gel electrophoresis; VA, velvet antler; WVAP, water velvet antler peptides.

The process of EVAP extraction was schematically shown in Figure 1A. Briefly, 1 g velvet antler (VA) powder was dissolved in 5 ml ddH_2_O (1:5), the solution pH adjusted to 2.0 with concentrated HCl, and then 0.01 g pepsin added. The solution was incubated at 37°C for 12 h. The enzyme was inactivated by boiling for 15 min. After cooling to room temperature (RT), pH of the solution was adjusted to 7.8–8.5 with 10 mM NaOH. Subsequently, 0.04 g trypsin was added, and the solution was incubated at 37°C for 1 h. The enzyme was inactivated by boiling for 15 min and then cooled down to RT. The EVAP solution was vacuum freeze-dried.

The process of water velvet antler peptides (WVAP) was described as follows. Briefly, 1 g VA powder was dissolved in 10 ml ddH_2_O (1:10), the solution was boiled for 0.5 h and then incubated at 37°C for 12 h.

### High Performance Liquid Chromatography Analysis of EVAP and WVAP

Isometric elution was performed with the mobile phase (40:60:0.05 ratio of acetonitrile, distilled water and trifluoroacetate) and a flow rate at 0.5 ml/min for 30 min. The column was flushed with mobile phase for 15 min and re-equilibrated for 15 min to the starting conditions for the next run. The UV detection acquisition wavelength was set at 220 nm and all measurements were performed at 30°C. The injection volume was 10 μl/time. Before being used, the mobile phase was filtered through a 0.45 μm membrane filter (Millipore, Milford, MA) and degassed using ultrasound.

### SDS-PAGE Electrophoresis of EVAP and WVAP

The EVAP and WVAP were separated *via* polyacrylamide SDS gel and stained with Coomassie blue. Decolor the gel with ethanol-glacial acetic acid and take pictures until the background of the gel was removed.

### Wound Creation and EVAP Treatment

Female SD rats, 6–8 weeks old, were purchased from Liaoning Changsheng Biotechnology Co., Ltd. (Benxi, China). All the animal experiment protocols and procedures were approved by the Animal Ethics Committee of Changchun Sci-Tech University (No. CKARI202003). The rats were anesthetized with 10% chloral hydrate (3 ml/kg), the dorsal hair was shaved, and then the skin wounds were created by cutting through full-thickness skin (diameter = 12 mm) without damaging the subcutaneous muscle tissue using a skin biopsy punches. EVAP solution was mixed with Matrigel (Corning, USA, used as a scaffold). The mixture of EVAP (100 μg/ml): Matrigel (1:1) was injected surrounding each wound (25 μl/injection × 4 injections) and topically applied (100 μl/wound) over the wound at the same time once every 7 days for 28 days. The wound healing status was recorded *via* photographing every seven days; the wound area was measured using the lasso tool of Photoshop (Adobe), which circled the wound area and calculated it based on the pixels. Twenty-eight days after treatment, the rats were sacrificed, and then the healed wound tissues were collected for later use; specifically, the healed skin tissue was divided into two parts along the central axis. One part (with immediately surrounding healthy tissue) was fixed in 10% formaldehyde solution used for histological experiments, and another part (without surrounding healthy tissue) was frozen at −80°C used for molecular biology experiments. PBS, epidermal growth factor (EGF, a growth factor that can accelerate reepithelialization and is usually used in wound healing) (22, 23), and WVAP, all mixed with Matrigel, served as the controls.

### Myofibroblast Transdifferentiation Induction *in vitro* and EVAP Treatment

Human dermal fibroblasts (HDFs) were seeded into a 24-well plate with a density at 50,000 cells/well for 24 h culture; then TGF-β1 (25 ng/ml) was added to induce myofibroblast transdifferentiation, at the same time, EVAP (25 μg/ml) was also added for another 24 h culture. Immunofluorescence (IF) staining was carried out to detect the expression of α-SMA. The related genes in the TGF-β signaling pathway, including TGF-β1, Smad2, p-Smad2, α-SMA, and collagen I, were analyzed *via* western blot analysis.

### Histology

The skin samples for histology were fixed in 10% formaldehyde solution, dehydrated by graded ethanol, and embedded in paraffin. The sections (4.0 μm) were cut and stained with hematoxylin and eosin (HE) and Masson, and then photographed under a Microscope (Precipoint M8, Germany). The number of skin appendages (hair follicles and sebaceous glands) was counted per 20 × high-power field (HPF) in the healed skin according to the image of HE staining; the percentage of collagen area (blue) was quantitated per HPF using Image-Pro Plus software in the healed skin according to the image of Masson staining.

For IF staining, paraffin sections or fixed-cells were incubated with primary antibodies overnight at 4°C and then incubated with second antibodies for 1 h at RT. DAPI (Beyotime, China) staining was carried out for nuclear visualization, and finally the sections were photographed under a Microscope (VOS M5000, USA); antibodies used in this study were list in Supplementary Table 1. The positive expression number of cells (DAPI^+^-target gene^+^) was quantified using Image-Pro Plus software.

### Quantitative Real-Time Polymerase Chain Reaction

Total RNA from healed skin was isolated using Trizol reagent (Invitrogen, USA). Then cDNA was synthesized from total RNA using cDNA Synthesis Kit (Takara, Japan) according to the manufacturer's protocol. The qRT-PCR analysis was performed using SYBR Green Master (Roche, Switzerland) in qTOWER ^3^G (Analykit Jena AG, Germany). GAPDH was used as the internal control. Primers were listed in Supplementary Table 2. All reactions were performed in triplicates.

### Western Blot Analysis

Total proteins were extracted from each sample using RIPA reagent (Invitrogen, USA) and then separated *via* polyacrylamide SDS gel and electrophoretically transferred onto polyvinylidene fluoride membranes (Millipore, MA). The membranes were then incubated with the primary antibodies (Supplementary Table 1) and subsequently the secondary antibody. The proteins were quantified using an ECL system. Western blot analyses were performed in triplicates, and the target protein bands were quantified using scanning densitometry, Image-J software, and normalized to the signal intensity of GAPDH. The relative density (RD) of protein bands was analyzed *via* ImageJ software.

### Statistical Analysis

All quantitative data are shown as the mean ± SD (*n* ≥ 3). Statistical analysis was conducted using Graphpad Prism software, and significant differences were evaluated using *t*-test and one-way analysis of variance. *P* < 0.05(^*^), <0.01(^**^), <0.001(^***^), or <0.0001(^****^) was considered statistically significant.

## Results

The SDS-PAGE electrophoresis was performed to determine the molecular weight of EVAP and WVAP components. There were all two protein bands detected both in EVAP and WVAP; however, the bands of EVAP lay between 10 and 26 kD, WVAP lay between 55 and 95 kD (Figure 1B). Besides, 14 uniformly distributed peaks were identified in EVAP through HPLC profiling, and the retention times of these peaks were between 11 and 23 min; however, one sharp peak and 10 flat peaks were identified in WVAP (Figure 1C).

### EVAP Increased the Healing Rate of Cutaneous Wounds in Rats

**Figure 2:**
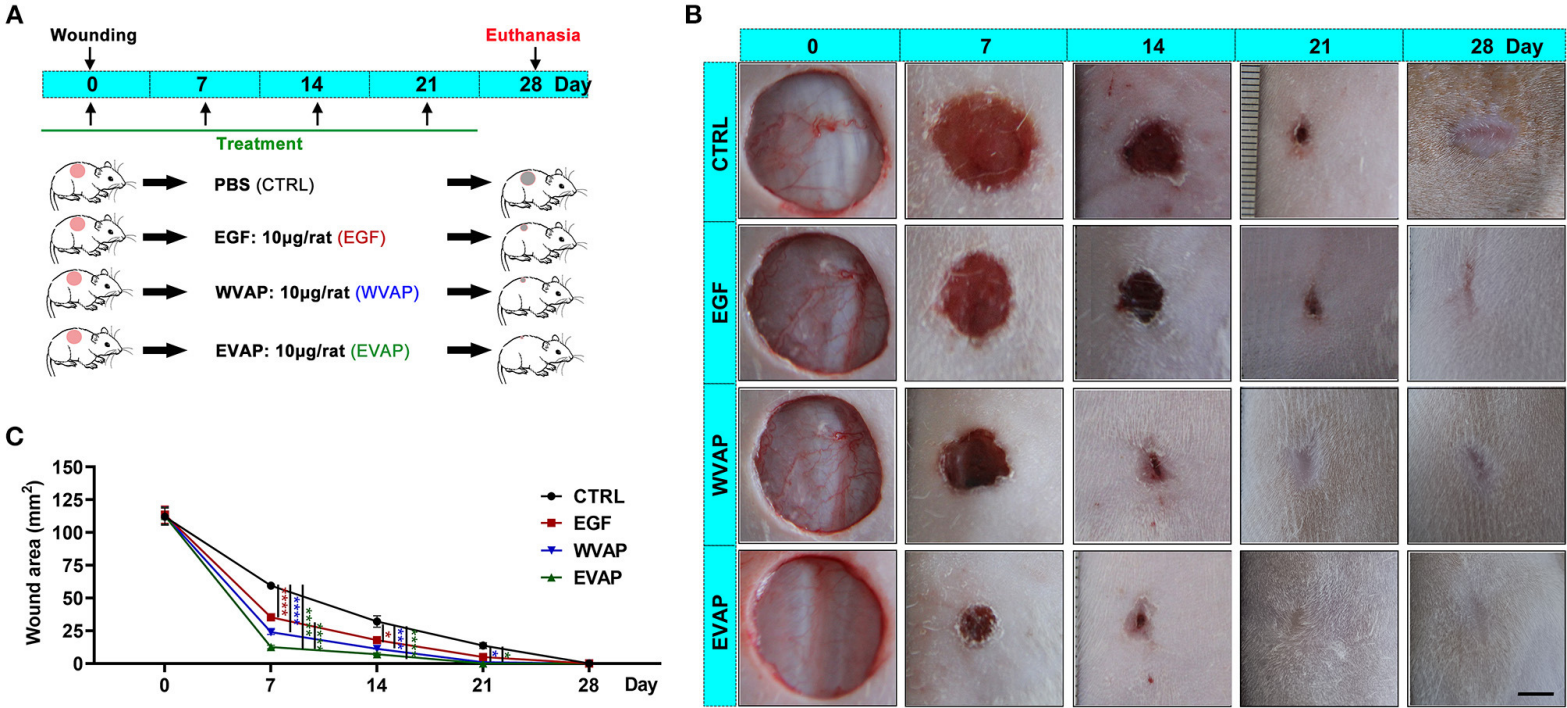
Effects of EVAP on healing rate of the full-thickness skin wound in rats. **(A)** Schematic drawing of experimental design. The dorsal hair was shaved, and then full-thickness skin wounds (diameter = 12 mm) were created on the shaved skin. EVAP solution was administered *via* both local injection surrounding each wound and topical application over the wound once every 7 days for 28 days. PBS, EGF, or WVAP served as controls. **(B)** Morphological changes of the wounds during the healing period; scale bar = 5 mm. **(C)** Quantitative evaluation of changes in wound area during the healing period. Note that the fastest wound healing rate and the smallest wound area occurred in the EVAP group compared to the control groups. *P < 0.05; ***P < 0.001; ****P < 0.0001; mean ± SD; *n* = 7. CTRL, control; EGF, epidermal growth factor; EVAP, enzymatically-digested velvet antler peptides; WVAP, water velvet antler peptides.

To evaluate the effects of EVAP on wound healing rate, we treated an equal amount of Matrigel mixed with either EVAP, WVAP, EGF, or PBS around the wounds (Figure 2A). The results showed that 7 days after the treatments, the wound areas (mm^2^) of all groups in order of size were CTRL (59.46 ± 3.27) > EGF (35.30 ± 3.72) > WVAP (23.95 ± 2.55) > EVAP (12.60 ± 1.93). Fourteen days after the treatments, the wound areas in the EVAP (7.12 ± 1.44), WVAP (11.16 ± 0.14), and EGF (17.86 ± 2.24) groups were significantly smaller than those of the CTRL group (15.20 ± 1.53) (*P* < 0.05; Figures 2B,C). Twenty-one days after the treatment, the wounds in the EVAP and WVAP groups were visibly closed, but those in the EGF (4.91 ± 0.80) and CTRL (13.66 ± 3.09) groups were not (Figures 2B,C), which were closed on 28 days after treatment with larger scar area than that of the EVAP group.

### EVAP Improved the Healing Quality of Cutaneous Wounds in Rats

**Figure 3:**
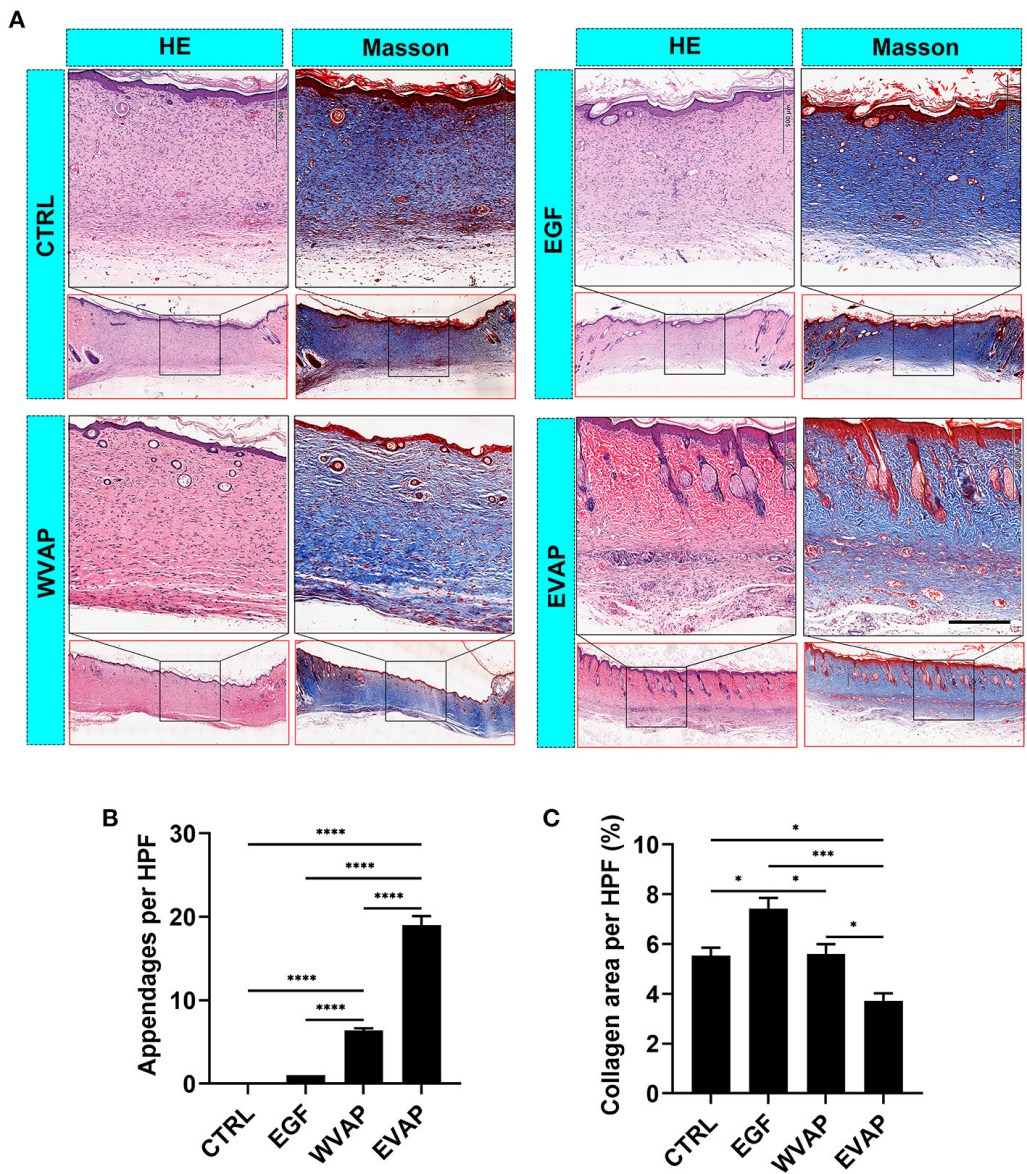
Effects of EVAP on healing quality of the wound tissue in model rats. **(A)** Histological sections of the healed skin stained either with HE or Masson (scale bar = 200 μm). **(B)** Number of skin appendages (hair follicles and sebaceous glands) in the healed skin per HPF. **(C)** Percentage of collagen area (blue) in the healed skin per HPF. Note that EVAP group had the highest number of cutaneous appendages but the least percent of collagen area compared to the control groups. *P < 0.05; ***P < 0.001; ****P < 0.0001; mean ± SD; *n* = 3. HE, hematoxylin and eosin; HPF, 20× high-power field.

Following the evaluation of the effects of EVAP on wound healing rate and the resultant scar area morphologically, we examined the healed tissue at the histological level. The results showed that 28-days after the treatments, healed skin in the EVAP group regenerated more appendages (hair follicles and sebaceous glands) than those in the CTRL, EGF, or WVAP group (all *P* < 0.0001; Figures 3A,B). Moreover, there was a significantly lower percent of collagen area in the healed skin being detected in the EVAP group than those in the CTRL (*P* < 0.05), EGF (*P* < 0.001), or WVAP (*P* < 0.05) group (Figures 3A,C). The collagens of healed skin in the EVAP group were found in a basketweave-like pattern, which was different from the bundle collagens in the EGF and CTRL groups (Figure 3A), suggesting that EVAP significantly improved the healing quality.

### EVAP Promoted Angiogenesis and Increased the Related Gene Expression in Healed Skin in Rats

**Figure 4:**
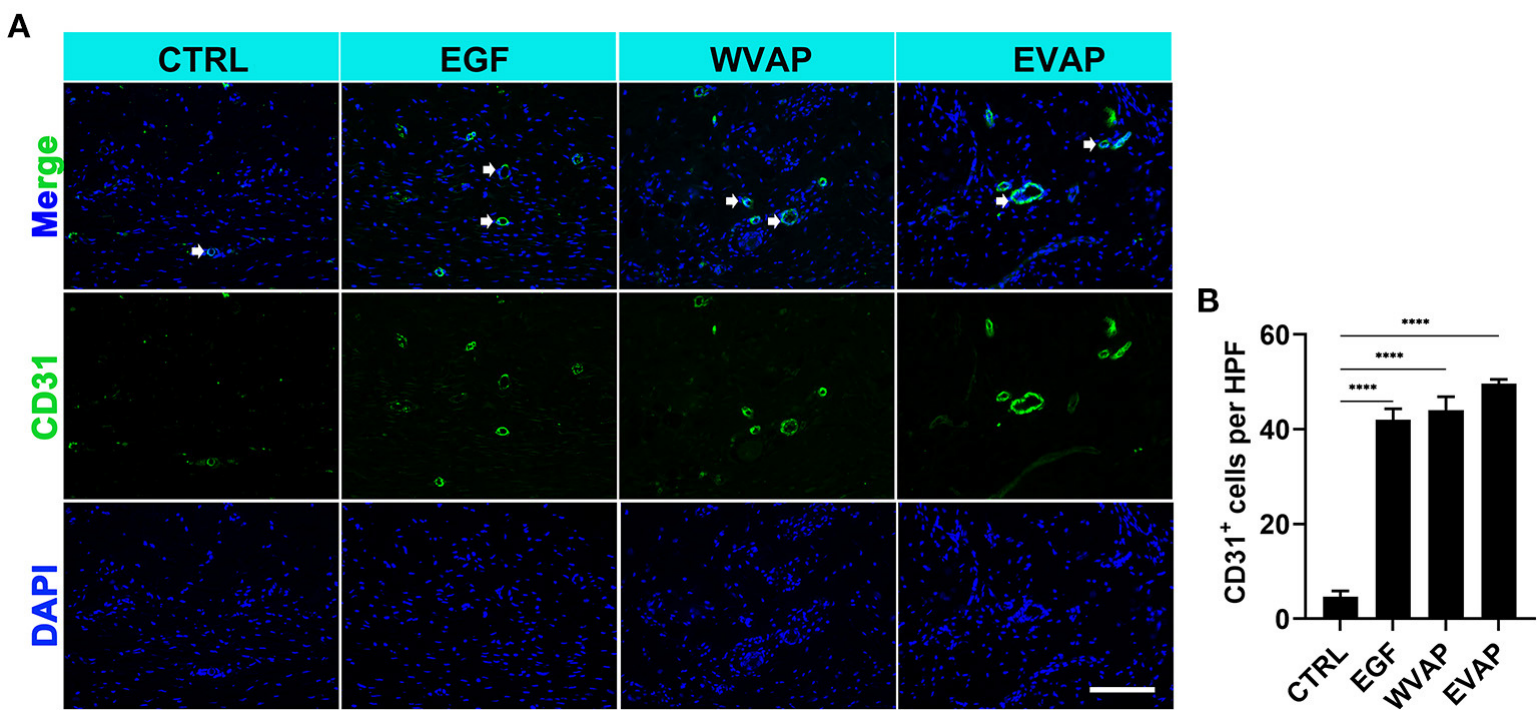
Effects of EVAP on blood vessel regeneration of the healed tissue in model rats. **(A)** CD31 staining on the histological sections (scale bar = 300 μm). **(B)** Quantification of CD31^+^ cells per HPF. Note that EVAP significantly increased the expression levels of CD31 compared to the control group. ****P < 0.00001; mean ± SD; *n* = 3. CD31, platelet and endothelial cell adhesion molecule 1.

**Figure 5:**
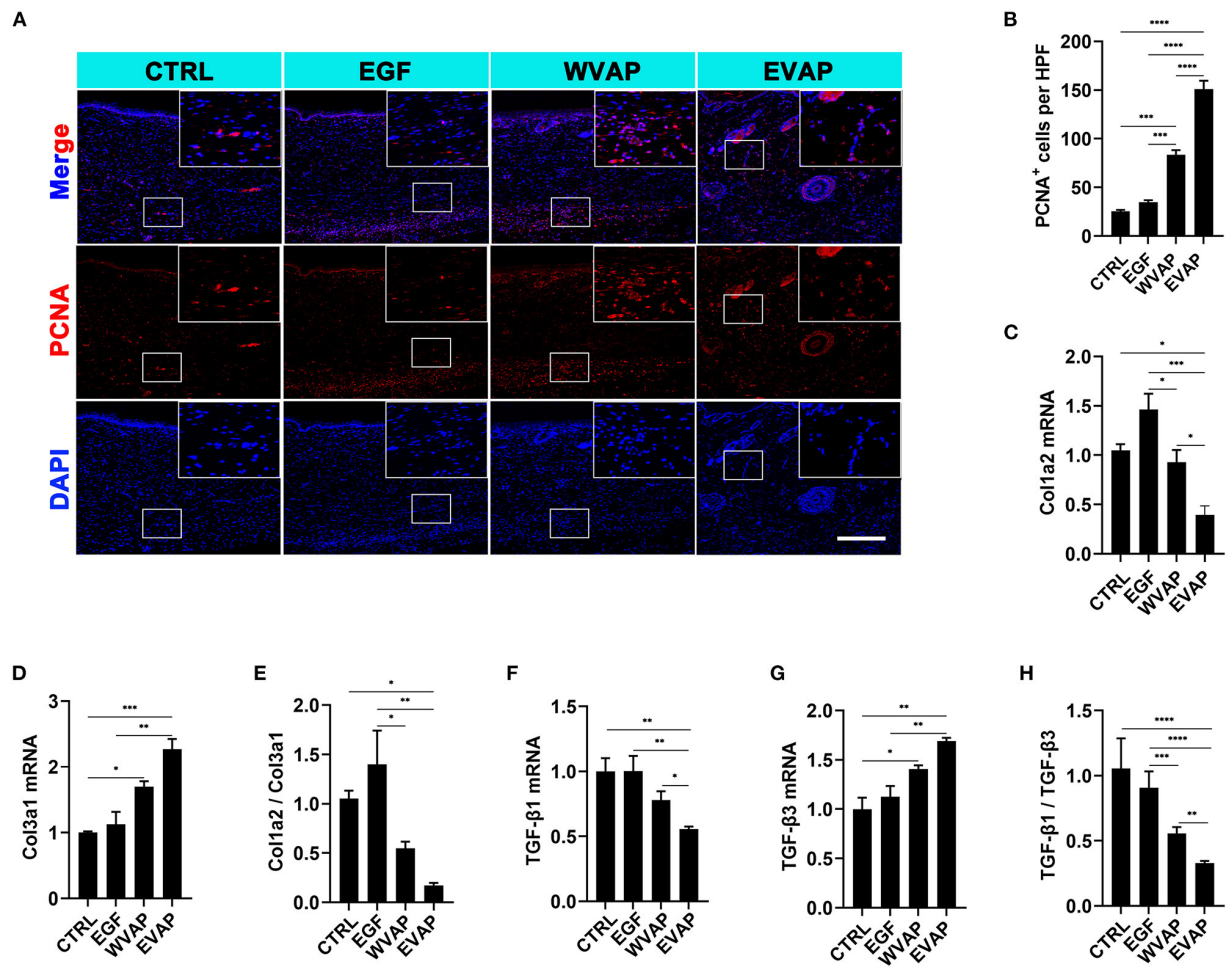
Effects of EVAP on cell proliferation and the expression levels of scar-related genes of the healed tissue in model rats. **(A)** PCNA staining on the histological sections (scale bar = 300 μm). **(B)** Quantification of PCNA^+^ cells per HPF. Note that EVAP significantly increased the expression levels of PCNA compared to the controls. **(C–H)** mRNA expression levels of Col1a2, Col3a1, TGF-β1, TGF-β3, and ratios of Col1a2/Col3a1 and TGF-β1/TGF-β3 in the healed skin. Note that genes pro-scar formation (Col1a2 and TGF-β1) were significantly down-regulated, whereas those anti-scar formation (Col3a1, TGF-β3, MMP1, and MMP3) up-regulated. *P < 0.05; **P < 0.01; ***P < 0.001; ****P < 0.0001; mean ± SD; *n* = 3. Col1a2, collagen type I α 2; Col3a1, collagen type III α 1; PCNA, proliferating cell nuclear antigen; TGF-β, transforming growth factor-β.

Next, we investigated the effects of EVAP on blood vessel regeneration of healed skins using IF. The results showed that 28-days after the treatments, the healed skin expressed more CD31 (a marker of vascular endothelial cells) in the EVAP group than in the CTRL group (*P* < 0.0001; Figures 4A,B). The expression level of PCNA (a marker of proliferating cells) of the healed skin in the EVAP group was also higher than in the CTRL, EGF, or WVAP group (all *P* < 0.0001; Figures 5A,B). These results suggest that EVAP promoted angiogenesis and cell proliferation in the healed tissue.

We also detected the mRNA levels for the genes relevant to regenerative wound healing, namely Col1a2, Col3a1, TGF-β1, and TGF-β3. Results showed that the healed skin in the EVAP group expressed lower Col1a2 and TGF-β1, higher Col3a1 and TGF-β3 than those in the CTRL, EGF, or WVAP group (*P* < 0.05; Figures 5C,D,F,G). Moreover, the ratios of Col1a2 to Col3a1 in the EVAP group were lower than those in the CTRL and EGF groups (*P* < 0.05, *P* < 0.01; Figure 5E), the ratios of TGF-β1 to TGF-β3 in the EVAP group were lower than those in the CTRL, EGF, and WVAP groups (*P* < 0.0001, *P* < 0.0001, *P* < 0.01; Figure 5H). Overall, our results suggest that the EVAP treatments have significantly improved wound healing quality in rats.

### EVAP Inhibited Myofibroblast Transdifferentiation

**Figure 6:**
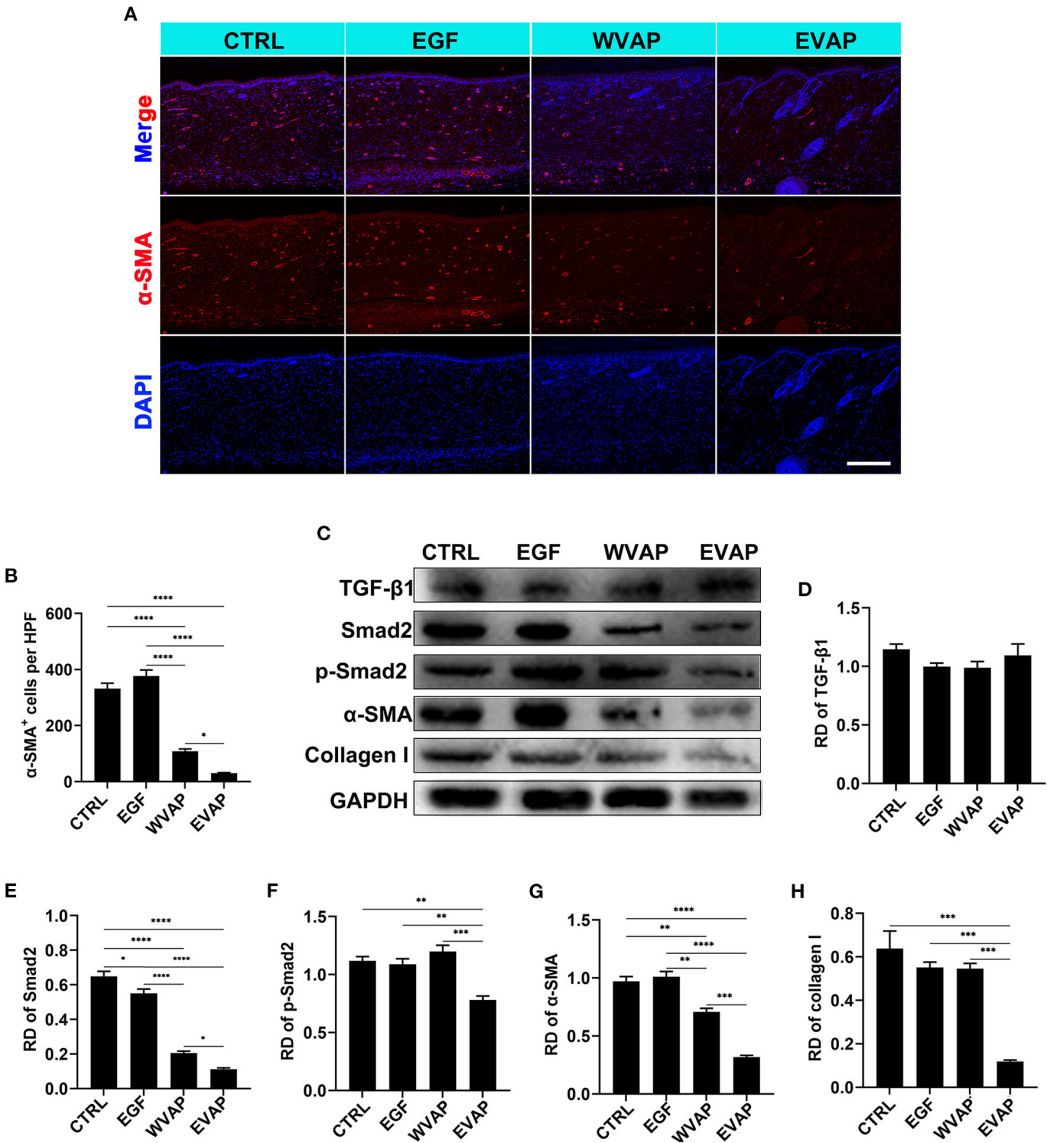
Effects of EVAP on myofibroblast transdifferentiation in the healed tissue of model rat*s*. **(A)** α-SMA staining on the histological sections (scale bar = 300 μm). **(B)** Quantification of α-SMA^+^ cells per HPF. **(C)** Protein expressions of TGF-β1, Smad2, p-Smad2 α-SMA, and collagen I in the healed skin. **(D–H)** RD of WB bands in **(B)**. Note that EVAP significantly inhibited the TGF-β signaling pathway *in vivo*. *P < 0.05; **P < 0.01; ***P < 0.001; ****P < 0.0001; mean ± SD; *n* = 3. RD, relative density; α-SMA, α-smooth muscle actin; WB, western blot.

α-SMA is the marker of myofibroblasts (2, 7). Thus, the expression of α-SMA in the healed skins were detected using IF. The results showed that the healed skin in the EVAP group expressed a significantly lower level of α-SMA than those in the CTRL (*P* < 0.0001), EGF (*P* < 0.0001), or WVAP (*P* < 0.05) group (Figures 6A,B).

**Figure 7:**
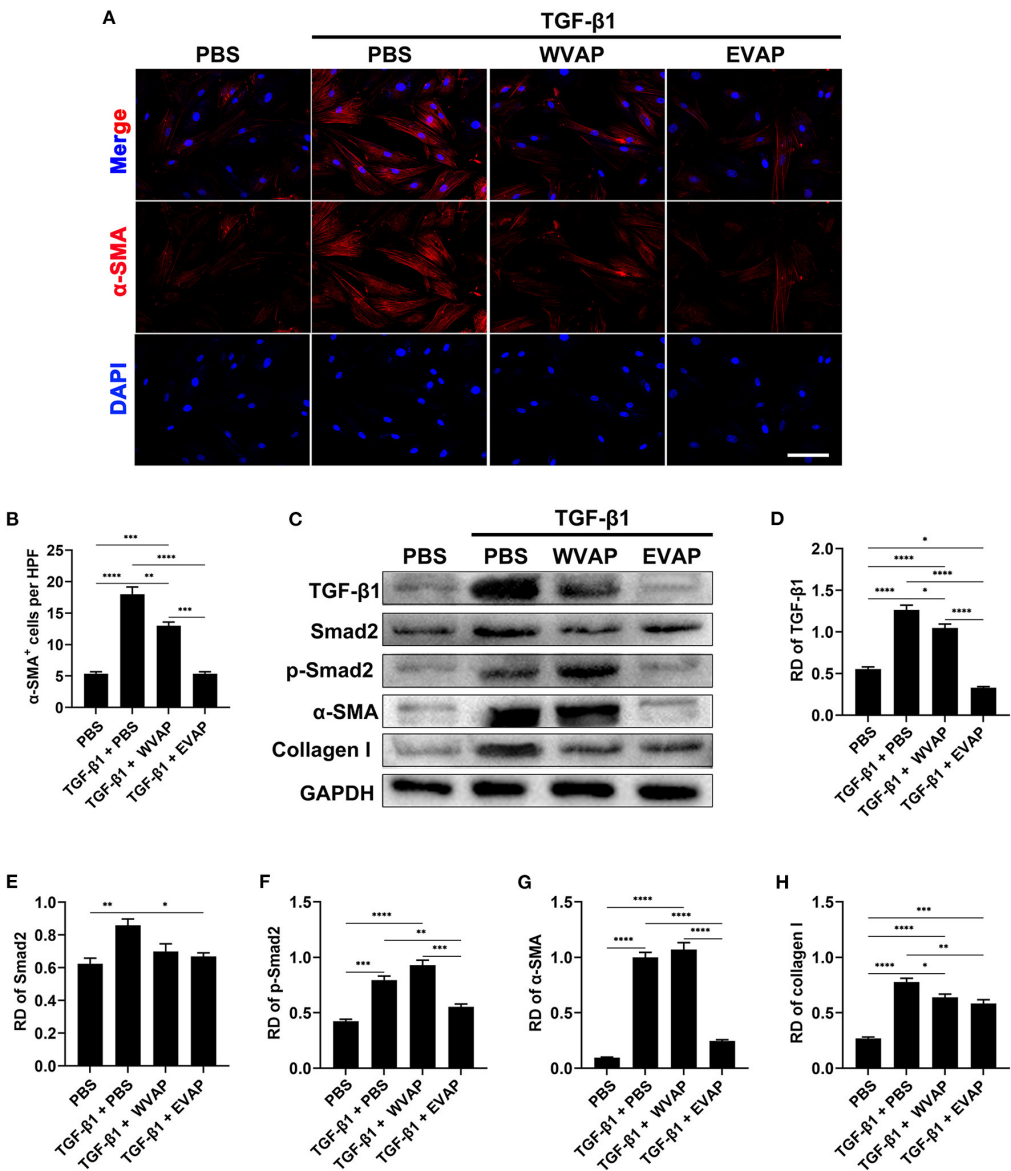
Effects of EVAP on myofibroblast transdifferentiation in the TGF-β1-induced HDFs. **(A)** α-SMA staining on the TGF-β1-induced HDFs (scale bar = 300 μm). **(B)** Quantification of α-SMA^+^ cells per HPF. **(C)** Protein expressions of TGF-β1, Smad2, p-Smad2 α-SMA and collagen I in the TGF-β1-induced HDFs. **(D–H)** RD of WB bands in **(B)**. Note that EVAP significantly inhibited the TGF-β signaling pathway *in vitro*. *P < 0.05; **P < 0.01; ***P < 0.001; mean ± SD; *n* = 3. HDFs, human dermal fibroblasts; RD, relative density.

To further reveal the possible mechanism underlying EVAP inhibition of myofibroblast transdifferentiation, we detected the expression levels of key point genes along the TGF-β signaling pathway, which is reported to play a critical role in myofibroblast transdifferentiation (24, 25). As expected, the results showed that the healed skin in the EVAP group expressed significantly lower levels of Smad2, p-Smad2, α-SMA, and collagen I than those in the CTRL, EGF, or WVAP group (*P* < 0.05; Figures 6C–H); however, the levels of TGF-β1 had no significant differences in these groups. To further verify these results, we carried out an *in vitro* study, in which EVAP was added to the HDFs (+TGF-β1) culture system to determine whether or not they could directly affect the expression levels of α-SMA. The results showed that EVAP treatment strongly inhibited the TGF-β1-induced expression level of α-SMA (Figures 7A,B). Moreover, the expression levels of TGF-β1, Smad2, p-Smad2, α-SMA, and collagen I in the TGF-β signaling pathway were all down-regulated by the EVAP treatment (Figures 7C–H). Overall, our results suggest that EVAP induced regenerative wound healing may be *via* inhibiting myofibroblast transdifferentiation, which could be at least partially *via* targeting the TGF-β signaling pathway.

## Discussion

Scar formation is generally the consequence of wound healing in mammals, caused by the excessive accumulation of collagen I. Myofibroblasts are known as collagen secreting cells (stronger than fibroblast) that have contractile ability and form microfilament bundles, which are the main contributors to scar formation (1, 3). Thus, it has been proposed that down-regulation of the formation of myofibroblasts is an effective way to prevent scar formation. WVAP-based therapies have been reported to alleviate fibrotic diseases effectively (19–21). However, no study has thus far focused on the effects of WVAP on wound healing and reduction of scar formation. To the best of our knowledge, this study is the first report that EVAP effectively enhanced the wound healing rate and improved the quality (regeneration of skin appendages and formation of the basketweave-like pattern of collagen fibers). Further examination showed that EVAP significantly suppressed the excessive myofibroblast transdifferentiation from fibroblasts and collagen I formation *in vivo* and *in vitro*, which may be achieved by down-regulating the TGF-β signaling pathway. We believe our study opens up a new avenue for preventing scar formation using EVAP in clinics.

Application of EVAP to treat full-skin-thickness wounds in rats in the present study was inspired by the phenomenon that the rapid and perfect cutaneous wound healing in deer (completion of a 10 cm wound healing can be achieved within 10 days) either at orthotopic (distal end of the pedicle) or at ectopic location (forehead), so long as closely associated with the antler tissue containing antler stem cells (26). In the previous studies, we used the antler stem cells to treat skin wounds in rats and found that the treatment could significantly accelerate wound healing rate and improve healing quality (27). Further study revealed that the therapeutic effects of antler stem cell treatment on wound healing were achieved through the paracrine pathway (28). Therefore, these previous studies have laid the foundation for formulating a cell-free therapy on regenerative wound healing in mammals and particularly in humans in clinics.

One drawback of using paracrine factors (conditional medium) of antler stem cells is lack of resources, even not to mention the formidable costs, thus not practical for clinical use. Nonetheless, we believe that these paracrine factors are extractable, and the extracted factors are still as effective as those collected from the conditional medium of these cells. If so, quantity is no longer a limiting factor for the development of therapeutics used for wound healing in clinics, as antlers are the fastest growing animal appendages, and growth rate can reach up to 2 cm/day and 30 kg within 60 days. Interestingly, WVAP has been found to be effective on treating some fibrotic diseases by targeting NF-κB and TGF-β signaling pathways through oral administration in some previous studies, such as carbon tetrachloride-induced liver fibrosis (19), pressure overload-induced cardiac fibrosis (20), and bleomycin-induced pulmonary fibrosis (21). These studies suggest that enzymatic digestion in the digestive tract may have helped to further potentiate antler extract containing factors. In the present study, we evaluated the effects of EVAP on wound healing rate and quality using a full-thickness skin wounded rat model and found that EVAP significantly reduced scar formation and promoted the regeneration of skin appendages, and the effects are significantly stronger than WVAP or EGF treatment.

During wound healing, dermal fibroblasts proliferate and migrate from a wound margin into the provisional matrix, where the activated fibroblasts can transdifferentiate into contractile myofibroblasts under the influence of the TGF-β signaling (4, 7). The transdifferentiated myofibroblasts display a “fibronexus,” which connects the intracellular microfilaments both to other myofibroblasts and surrounding extracellular matrix thereby mediating contraction (29). These indicate myofibroblasts plays an important role in promoting wound healing rate by 1) stimulating wound contraction to effectively reduce the wound size, 2) producing sufficient ECM to support granulation tissue formation. However, the continued transdifferentiation of myofibroblasts causes excessive accumulation of ECM and continued contraction, which leads to continued thickening of collagen fiber bundles and eventually results in scar formation (4, 7). Therefore, inhibition of myofibroblast transdifferentiation during wound healing is required to prevent myofibroblasts' accumulation, rather than taking remedial measures after scar formation. In this study, we observed that the expression of α-SMA and collagen I in the EVAP group was significantly lower compared with that in the control groups both *in vivo* and *in vitro*, suggesting that the ability of EVAP to improve wound healing quality including reduction of scar size may be achieved by inhibiting myofibroblast transdifferentiation.

It has been reported that TGF-β1 signaling is an essential regulatory factor in stimulating transdifferentiation of fibroblasts into myofibroblasts (2, 6, 30). Once the dermal fibroblasts of an open wound tissue exposed to TGF-β1, those cells would proliferate and migrate from a wound margin into the provisional matrix at sites of injury. The activated fibroblast can trigger formation of contractile myofibroblasts characterized by α-SMA expression (4, 7). As expected, in the present study, we demonstrated that EVAP significantly reduced the expression levels of the TGF-β signaling pathway related genes, including TGF-β1, Smad2, p-Smad2, α-SMA, and collagen I, in the healed skin *in vivo* and in the TGF-β1 induced HDFs *in vitro*, which was consistent with Zhao et al. (20), who reported that the sVAP32 (a component of antler extracts) interacts with TGF-β1 receptors and disrupts the TGF-β signaling pathway. However, the effects of EVAP on improving wound healing quality may not be caused just by a single factor, but rather by a combination of factors in the EVAP.

**Figure 8:**
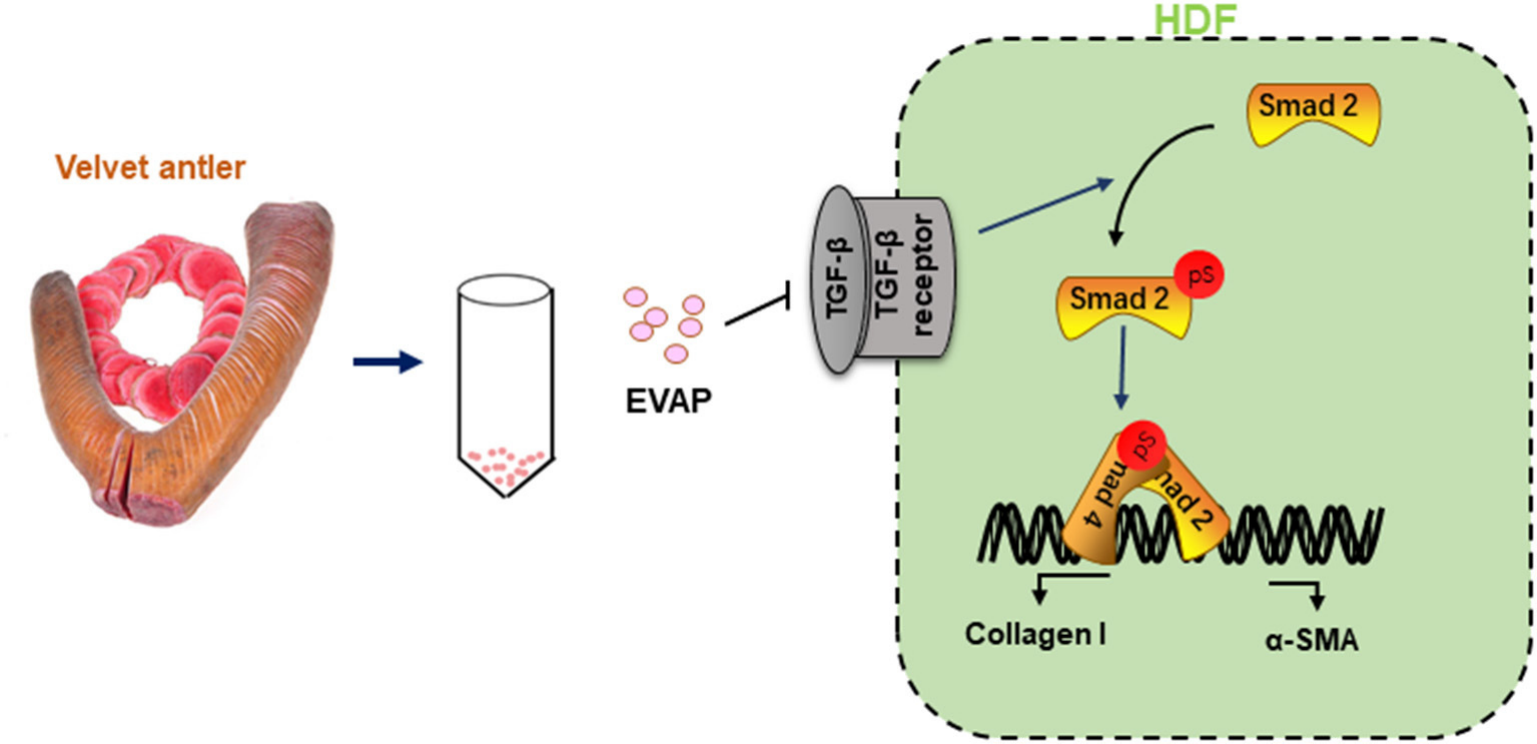
Reduction of scar formation with the treatment of EVAP may be through inhibiting the TGF-β signaling pathway. After wounding, expression level of TGF-β1 rises rapidly, which phosphorylates Smad2 to initiate the TGF-β1 signaling pathway. Activated TGF-β1 pathway stimulates the transdifferentiation of HDFs to myofibroblasts. Continuous myofibroblast transdifferentiation causes excessive collagen production and thus scar formation, and was effectively reduced through inhibition of the TGF-β signaling pathway and thus transdifferentiation of myofibroblasts with EVAP application.

In summary, this study is the first report that EVAP effectively stimulated scarless wound healing, reduced the degree of myofibroblast transdifferentiation and excessive collagen I accumulation. Furthermore, these effects of EVAP are highly likely achieved *via* inhibiting the TGF-β signaling pathway (Figure 8). Overall, we believe our study has opened a new avenue for the development of evidence-based therapeutics using traditional medicine materials and provided a new strategy for the clinical treatment of skin scar formation.

## Data Availability Statement

The original contributions presented in the study are included in the article/Supplementary Material, further inquiries can be directed to the corresponding author/s.

## Ethics Statement

The animal study was reviewed and approved by Animal Ethics Committee of Changchun Sci-Tech University.

## Author Contributions

CL and GZ: conceptualization, writing, review, and editing. DW and GZ: performance of the animal experiments. HS, LS, and JL: performance of cell experiments. JR: histology. LS and SW: validation and analyze. GZ: data curation. HZ and MY: preparation of enzymatically-digested velvet antler peptides. All authors contributed to the article and approved the submitted version.

## Funding

This research was funded by the National Natural Science Foundation of China (No. U20A20403), the Strategic Priority Research Program of the Chinese Academy of Sciences (No. XDA16010105), and the Project of Livestock and Poultry Resources Development and Utilization of Jilin Province.

## Conflict of Interest

The authors declare that the research was conducted in the absence of any commercial or financial relationships that could be construed as a potential conflict of interest.

## Publisher's Note

All claims expressed in this article are solely those of the authors and do not necessarily represent those of their affiliated organizations, or those of the publisher, the editors and the reviewers. Any product that may be evaluated in this article, or claim that may be made by its manufacturer, is not guaranteed or endorsed by the publisher.
