# Supplementary material for: Velvet Antler Peptides Reduce Scarring via Inhibiting the TGF-β Signaling Pathway During Wound Healing
Source: Front Med (Lausanne). 2022 Jan 21;8:799789. doi: 10.3389/fmed.2021.799789 (PMC8814364; doi:10.3389/fmed.2021.799789)
Supplement: Supplementary file 1 [file Data_Sheet_1.docx]

Supplementary Material

**Table S1** Antibodis

| **Antibody** | **Company** | **Catalog number** | **Dilution** |
| --- | --- | --- | --- |
| PCNA | ProteinTech, China | 60097-1-Ig | 1:500 (IF) |
| TGF-β1 | Bioss, China | bsm-33345M | 1:2000 (WB) |
| α-SMA | ProteinTech, China | 14395-1-AP | 1:200 (IF); 1:3000 (WB) |
| Smad2 | Beyotome, China | AF1300 | 1:2000 (WB) |
| p-Smad2 | Bioss, China | bs-7464R | 1:2000 (WB) |
| Collagen I | ProteinTech, China | 14695-1-AP | 1:3000 (WB) |
| CD31 | Abcom, UK | ab119339 | 1:300 (IF) |
| IgG-AF488 | ProteinTech, China | SA00013-1 | 1:50 (IF) |
| IgG-Cy3 | ProteinTech, China | SA00009-2 | 1:50 (IF) |
| GAPDH | Bioss, China | bs-13282R | 1:2000 (WB) |

**Table S2** Primers

|  | | qRT-PCR primers |
| --- | --- | --- |
| Collagen Ⅰ | F | 5’-GTCCTATGGCTATGATGAGAAATC-3’ |
|  | R | 5´-CACCATCCAAACCACTGAAAC-3’ |
| Collagen Ⅲ | F | 5´-AGCCACCTTGGTCAGTCCTA-3’ |
|  | R | 5´-GTGTAGAAGGCTGTGGGCAT-3’ |
| TGFβ1 | F | 5´-CACCTGCAAGACCATCGACA-3’ |
|  | R | 5´- GCATAGTAGTCCGCTTCGGG-3’ |
| TGFβ3 | F | 5’-AGAATCCCAGCGCTACAAGG-3’ |
|  | R | 5’-GTCCCCTAATGGCTTCCACC-3’ |
| GAPDH | F | TGCCCCCATGTTTGTGATG |
|  | R | TGTGGTCATGAGCCCTTCC |
